# The Schoolteachers' Version of the Stress and Anxiety to Viral Epidemics-9 (SAVE-9) Scale for Assessing Stress and Anxiety During the COVID-19 Pandemic

**DOI:** 10.3389/fpsyt.2021.712670

**Published:** 2021-08-11

**Authors:** Soyoung Yoo, Jihoon Lee, Gawon Ju, Sangha Lee, Sooyeon Suh, Seockhoon Chung

**Affiliations:** ^1^Department of Convergence Medicine, University of Ulsan College of Medicine, Seoul, South Korea; ^2^Department of Psychiatry, Asan Medical Center, University of Ulsan College of Medicine, Seoul, South Korea; ^3^Department of Psychiatry, Chungbuk National University Hospital, Cheongju-si, South Korea; ^4^Department of Psychiatry, Ajou University School of Medicine, Suwon, South Korea; ^5^Department of Psychology, Sungshin Women's University, Seoul, South Korea

**Keywords:** stress, psychological, teacher, COVID-19, anxiety

## Abstract

This study aimed to validate the schoolteachers' version of the Stress and Anxiety to Viral Epidemics-9 (SAVE-9) scale. This scale assessed the work-related stress and anxiety response of schoolteachers to the COVID-19 pandemic. A total of 400 schoolteachers participated in an online survey between March 4 and 15, 2021. The survey questionnaire included the schoolteachers' version of the SAVE-9, Patient Health Questionnaire-9 (PHQ-9), and Generalized Anxiety Disorders-7 (GAD-7) scales. A scree test and parallel analysis suggested a single-factor structure model for the schoolteachers' version of the SAVE-9 scale (real-data eigenvalue = 68.89, 95th percentile of the random eigenvalues = 27.56). The SAVE-9 scale showed good internal consistency (Cronbach's alpha = 0.853) and good convergent validity with GAD-7 (rho = 0.545, *p* < 0.001) and PHQ-9 (rho = 0.434, *p* < 0.001) scale scores. This, schoolteachers' version of the SAVE-9 scale is a reliable and valid rating scale that can be applied to teachers in a pandemic situation.

## Introduction

In 2020, people experienced psychological issues such as depression, anxiety, and post-traumatic stress resulting from the coronavirus disease 2019 (COVID-19) pandemic and the subsequent economic crises. COVID-19-related lockdowns, social distancing, and quarantine disrupted their daily lives, business dealings, and interpersonal relationships (1). Although COVID-19 vaccines have been developed and available since December 2020^1^, 185,291,530 confirmed cases and 4,010,834 deaths have been recorded globally. These are staggering and tragic numbers^2^.

School environments were also severely disrupted and changed rapidly during the pandemic^3^. This resulted in schoolteachers experiencing high levels of stress or burnout (2, 3). When schools reopened, teachers who were not trained in infection control had the additional responsibility of preventing viral spread. This occurred by checking students' temperatures or rating their suspected symptoms. Moreover, they were required to direct students to wear masks and practice social distancing while at school. These tasks most likely imposed a severe burden on schoolteachers. Even when schools were closed owing to the lockdown, schoolteachers were required to adapt to a new educational environment and prepare for online education (4). They monitored students' academic abilities and supervised their progress while providing online classes. They also assisted in overcoming children's obstacles to learning (5) such as digital education inequity, absence of parents while students remained at home, and the lack of in-person teacher-student-parent relationships.

Reportedly, teachers had symptoms of stress, anxiety, and depression during the pandemic. This was especially evident among female teachers and those who had children (6). Their age, job stability, level of education, information sources, and level of apprehension also influenced their symptoms (2, 7). They experienced severe burnout (8), and their quality of life was hampered by work overload, feelings of uncertainty, and/or fear (9). Teachers played a key role in the educational environment during this pandemic. However, little research has been conducted focusing on their mental health, though they served as the forgotten frontline heroes of the COVID-19 pandemic (10). Further, to the best of our knowledge, there is no developed rating scale that can measure the anxiety and/or stress of teachers in response to specific viral epidemics. Therefore, we have attempted to develop the schoolteachers' version of the anxiety assessment scale using the SAVE-9 scale that has been developed for healthcare workers.

During the COVID-19 pandemic, healthcare workers, public workers, and schoolteachers cared for others at risk of COVID-19. They played roles in the prevention of viral spread, experienced severe psychological distress, and endured work-related burnout. They had to avoid becoming infected themselves and had to prevent the spread of viral infection to their family and co-workers. Therefore, it is important to develop epidemic-specific psychological assessment tools for the schoolteachers affected by the current pandemic. We recently developed a new rating scale, the Stress and Anxiety to Viral Epidemics-9 (SAVE-9) scale, to assess work-related stress and anxiety among healthcare workers in response to the COVID-19 pandemic (11). The scale was originally developed for frontline healthcare workers caring for patients during the COVID-19 pandemic. However, schoolteachers also were placed in a situation similar to that of healthcare workers. They cared for students and were also responsible for infection control in schools while being at risk of infection from students. Therefore, to address their mental health, it is important to assess the anxiety or work-related stress of schoolteachers during this pandemic situation. This study explored the validity of the schoolteachers' version of the SAVE-9 scale for assessing work-related stress among schoolteachers during the current COVID-19 pandemic.

## Materials and Methods

### Participants and Procedures

We enrolled schoolteachers working in elementary, middle, and high schools in various regions in Korea. This study was conducted via an online survey administered by a professional online survey company, EMBRAIN^4^, during March 4–15, 2021. The surveys were completed anonymously. No personal information was gathered. The study protocol was approved and the requirement for written informed consent for participation was waived by the Institutional Review Board of Asan Medical Center (2021-0098). The participants voluntarily responded to this questionnaire. We used only the responses of participants who agreed to provide their data for statistical analysis.

### Symptom Assessment

#### SAVE-9 Scale

The main objective of this study was to develop and validate the schoolteachers' version of the SAVE-9 scale. The SAVE-9 was originally developed to assess the mental health of healthcare workers' during the COVID-19 pandemic (11). The scale comprises nine items that considers two factors: (1) anxiety response to a viral epidemic and (2) work-related stress associated with a viral epidemic. To modify this SAVE-9 scale for schoolteachers, item 7 that stated “After this experience, do you think you will avoid treating patients with viral illnesses?” was changed to “Do you think you will avoid teaching children who have had viral illnesses.” The participants were asked to rate their responses on a five-point Likert scale ranging from 0 (never) to 4 (always)^5^.

#### Patient Health Questionnaire-9

The Patient Health Questionnaire-9 (PHQ-9) scale is a self-administered questionnaire that is used to assess depressive symptoms. Respondents can rate their symptoms for each of the nine items on a three-point Likert scale (0 = not at all to 3 = nearly every day). The total score of the PHQ-9 scale ranges from 0 to 27. The higher scores reflect more severe degrees of depressive symptoms (12). The present study defined the cut-off score for depression as ≥ 10.

#### Generalized Anxiety Disorder-7

The Generalized Anxiety Disorder-7 (GAD-7) scale is a self-administered questionnaire used to assess general anxiety. Respondents can rate their anxiety symptoms for each of the seven items on a three-point Likert scale (0 = not at all to 3 = nearly every day). The total score of the GAD-7 scale ranges from 0 to 21. The higher scores reflect more severe degrees of anxiety symptoms (13). The present study defined a score of 5 as the cut-off to identify cases with at least mild anxiety.

### Statistical Analysis

We conducted a principal component analysis (PCA) with varimax rotation to explore the factor structure of the schoolteachers' version of the SAVE-9 scale. Prior to conducting this analysis, we checked the normality assumption of each item based on its skewness and kurtosis, with an acceptable limit of range of ± 2 (14). We also evaluated the data suitability and sampling adequacy using Kaiser–Meyer–Olkin values and Bartlett's tests of sphericity. A scree test and parallel analysis (15–17), according to a minimum rank factor analysis (MRFA), with a 95th percentile threshold based on a polychoric correlations matrix was conducted to determine the number of factors to retain using the FACTOR 10.10.03. program (18). To define the factor structure, we compared the explained real-data eigenvalues with the 95th percentile of random eigenvalues. We decided where the real-data eigenvalues exceeded the 95th percentile of random eigenvalues. The reliability and internal consistency of the factors were examined using Cronbach's alpha and McDonald's Omega coefficients to verify the dimensionality of the schoolteacher's version of the SAVE-9 scale. To investigate the psychometric properties, independent *t*-tests were conducted using IBM SPSS Statistics for Windows, version 21.0. We assessed whether the SAVE-9 scale score differed significantly between groups based on sex (male vs. female), age (juniors vs. seniors), marital status (single vs. married), caring for infected students (yes vs. no), and being quarantined (yes vs. no). To determine the convergent validity, we performed a Spearman correlation analysis of the SAVE-9 scale score with age, years of employment, PHQ-9 scores, and GAD-7 scores, since the PHQ-9 and GAD-7 scales' scores did not show normal distributions. Finally, to define the appropriate cut-off score of the SAVE-9 scale consistent with the severity of the generalized anxiety (GAD-7 score), we conducted a receiver operating characteristic (ROC) analysis.

## Results

A total of 400 teachers participated in this study. They were from various regions in Korea, including Seoul (*N* = 78, 19.5%), Pusan (*N* = 28, 7.0%), Daegu (*N* = 28, 7.0%), Daejeon (*N* = 8, 2.0%), Gwangju (*N* = 10, 2.5%), Chungcheong Province (*N* = 34, 8.5%), Jeolla Province (*N* = 31, 7.7%), Gyeongsang Province (*n* = 49, 12.3%), Gangwon Province (*N* = 13, 3.3%), and Jeju Province (*N* = 4, 1.0%). Table 1 shows the demographic characteristics of the participants. Out of all the participants, 270 (67.5%) were female and the others were male; 177 (44.3%) were single; and 57 (14.2%) had experienced or had been treated for depression, anxiety, or insomnia. Their mean duration of employment was 11.8 ± 8.6 years. Among the participants, 71 (17.7%) had cared for students with confirmed COVID-19, 57 (14.2%) had been quarantined due to COVID-19, and 1 (0.3%) had developed COVID-19.

**Table 1:** Demographic characteristics of the participants (*N* = 400).

**Variables**	**Mean ± SD, *N* (%)**
Teachers	
Elementary school	140 (35.0%)
Middle school	130 (32.5%)
High school	130 (32.5%)
Sex (female)	270 (67.5%)
Age (years)	
20–29	75 (18.8%)
30–39	161 (40.3%)
40–49	112 (28.0%)
50–59	42 (10.5%)
60–69	10 (2.5%)
Marital status	
Single	177 (44.3%)
Married	223 (55.7%)
Years of employment	11.8 ± 8.6
COVID-19-related questions	
Did you care for children who have had viral illnesses? (Yes)	71 (17.7%)
Were you quarantined owing to exposure to COVID-19? (Yes)	57 (14.2%)
Were you positive for COVID-19? (Yes)	1 (0.3%)
Psychiatric history	
Have you ever experienced or were you treated for depression, anxiety, or insomnia? (Yes)	57 (14.2%)
Do you think you are currently depressed or anxious, or do you need help for your mood? (Yes)	44 (11.0%)

*COVID-19, coronavirus disease 2019; SD, standard deviation*.

### Factor Structure of the Schoolteacher's Version of the SAVE-9 Scale

Assessment of the normality assumption showed that the distributions of all nine items were within normal limits (Table 2). The data were suitable, and the sampling was adequate for PCA (Bartlett's test of sphericity, *p* < 0.001; Kaiser–Meyer–Olkin = 0.897). To explore the factor structure of the schoolteacher's version of the SAVE-9 scale, a PCA was conducted, and a two-factor model was explored based on the eigenvalue plot (first factor eigenvalue = 4.337, second factor eigenvalue = 1.054), which considered two factors: (1) Factor I: anxiety regarding the viral epidemic (items 1, 2, 3, 4, and 8) and (2) Factor II: work-related stress associated with the viral epidemic (items 5, 6, 7, and 9) (Table 2). However, the results of the scree test and parallel analysis using MRFA extraction and polychoric correlation (that was conducted to identify the adequate number of factors for the scale) suggested a single-factor structure model (real-data eigenvalue = 68.89, 95th percentile of random eigenvalue = 27.56) for the schoolteacher's version of the SAVE-9 scale. Therefore, we adopted the single-structure model.

**Table 2:** Factor structure of the schoolteachers' version of the SAVE-9 scale and factor loadings.

**Items**	**Responses**					**Mean ± SD**	**Skewness**	**Kurtosis**	**Factor loading**
	**0**	**1**	**2**	**3**	**4**				
1. Are you afraid the virus outbreak will continue indefinitely?	0.5%	5.0%	11.7%	56.5%	26.3%	3.03 ± 0.79	−0.903	1.187	0.509
2. Are you afraid your health will worsen because of the virus?	0.5%	6.3%	19.3%	49.7%	24.2%	2.91 ± 0.85	−0.637	0.194	0.626
3. Are you worried that you might get infected?	1.0%	8.3%	17.5%	47.2%	26.0%	2.89 ± 0.92	−0.732	0.159	0.708
4. Are you more sensitive toward minor physical symptoms than usual?	1.5%	10.5%	15.2%	45.0%	27.8%	2.87 ± 0.99	−0.786	0.069	0.681
5. Are you worried that others might avoid you even after the infection risk has been minimized?	9.8%	26.5%	24.7%	24.5%	14.5%	2.08 ± 1.22	0.019	−1.011	0.611
6. Do you feel skeptical about your job after going through this experience?	6.5%	26.8%	28.7%	22.3%	15.8%	2.14 ± 1.17	0.090	−0.928	0.695
7. Do you think you will avoid teaching children who have had viral illnesses?	8.3%	21.5%	24.2%	35.5%	10.5%	2.19 ± 1.14	−0.268	−0.812	0.565
8. Do you worry your family or friends may become infected because of you?	2.0%	6.8%	20.5%	43.8%	27.0%	2.87 ± 0.95	−0.754	0.296	0.759
9. Do you think that your colleagues would have more work to do due to your absence from a possible quarantine and might blame you?	4.8%	10.7%	19.8%	43.8%	21.0%	2.66 ± 1.07	−0.732	−0.048	0.765

*SAVE-9, Stress and Anxiety to Viral Epidemics-9 items; SD, standard deviation*.

*0 = never; 1 = rarely; 2 = sometimes; 3 = often; 4 = always*.

### Reliability of the Scores and Evidence Based on Relationships With Other Variables

The schoolteachers' version of the SAVE-9 scale showed good internal consistency (Cronbach's alpha = 0.853 and McDonald's Omega = 0.850). The Spearman correlation coefficient showed significant correlations between the SAVE-9, GAD-7 (rho = 0.545, *p* < 0.001), and PHQ-9 (rho = 0.434, *p* < 0.001) scale scores. The SAVE-9 scale score was significantly higher among healthcare workers who were rated as having generalized anxiety [GAD-7 ≥ 10, *t*_(398)_ = 10.155, *p* < 0.001] and depression [PHQ-9 ≥10, *t*_(398)_ = 7.896, *p* < 0.001] than among those who did not. The SAVE-9 scale score was significantly higher for women than for men [*t*_(398)_ = 4.063, *p* < 0.0001]; however, it did not differ significantly according to marital status [single vs. married, *t*_(398)_ = 1.140, *p* = 0.255], age [junior 20–39 years vs. senior over 40 years, *t*_(398)_ = 0.923, *p* = 0.356], experience caring for infected students [*t*_(393)_ = 1.660, *p* = 0.098], or experience being quarantined [*t*_(398)_ = 0.773, *p* = 0.440].

### Cut-Off Score for the SAVE-9 Scale

To explore the appropriate cut-off score of the SAVE-9 scale that corresponded to at least mild generalized anxiety, we conducted an ROC analysis using a GAD-7 scale score of 5. The ROC analysis revealed that the appropriate SAVE-9 cut-off score was ≥ 22 (area under the curve [AUC] = 0.729, sensitivity = 0.79, specificity = 0.54).

## Discussion

The results of this study showed that the schoolteacher's version of the SAVE-9 scale was reliable, with good internal consistency. We adopted the single-factor model of the SAVE-9 scale. Moreover, the schoolteachers' version of the SAVE-9 scale showed good convergent validity with a pre-existing anxiety measurement tool (the GAD-7 scale). It exhibited a threshold score of 22 indicating at least mild generalized anxiety (GAD-7 scale score ≥5).

A schoolteacher's role in this pandemic is like that of a healthcare worker. The teacher must care for his or her students just as the healthcare worker must care for his or her patients. For this sample of schoolteachers, we adopted a single-factor model based on the results of the parallel analysis, though PCA suggested a two-factor model. Parallel analysis was used to determine the number of components to retain from PCA (19). Parallel analysis is conducted based on the rationale of multiplying the total number of variable loadings by the significance level to provide an empirical estimate of the 95th percentile. We can also estimate by checking where the real-data eigenvalues exceeded the 95th percentile of the random eigenvalues. The schoolteachers' version of the SAVE-9 scale showed good internal consistency and convergent validity. In our previous sample of healthcare workers (11), items related to work-related stress (items 6, 7, and 9) were clustered separately, though items 5, 6, 7, and 9 were clustered in this sample in PCA. This difference may be due to differences in the working environments between healthcare workers and schoolteachers, the time frame of the survey, or the sampling variability.

The factor loading values of items 1 and 7 in this sample were 0.509 and 0.565, respectively. Items are generally accepted when their factor loading values are >0.6 (20). However, items with values >0.5 may be acceptable if the consistency of the scale is good (21). Thus, we adopted all the items in the final model.

The appropriate cut-off score of the schoolteachers' version of the SAVE-9 scale was ≥22 to indicate at least a mild degree of generalized anxiety (GAD-7 ≥5) (AUC = 0.729, sensitivity = 0.79, specificity = 0.54). These results are consistent with those reported for the original SAVE-9 scale (11). We originally developed the SAVE-9 scale as a viral epidemic-specific anxiety rating scale especially for workers addressing or caring for people at high risk of infection in a pandemic. We intended to identify a mild degree of anxiety response to the viral epidemic to screen for healthcare workers requiring psychological support during the current pandemic. Similarly, we defined a cut-off score for the schoolteachers' version of the SAVE-9 scale. It corresponded to a GAD-7 score of ≥5 (mild degree) to screen for schoolteachers with at least mild anxiety regarding the current viral epidemic. Thus, the schoolteachers' version of the SAVE-9 scale can be applied as it showed good validity in the educational setting.

This study had several limitations. First, the survey was conducted via an anonymous online survey as it was not easy to interview the participants face-to-face during the current pandemic situation. Though we used the services of a professional survey company, the nature of the survey may have introduced errors. Second, the survey was conducted about 1 year after the outbreak. Thus, the teachers might have adapted to their situations, developed online education skills, and cared for students during the pandemic in 2020. This may influence the validity of the schoolteachers' version of the SAVE-9 scale. In this study, we could not collect the data of participants' race, income, education, or socioeconomic status. The status of the pandemic in Korea might be different from that in other countries in which the pandemic is severe. The school or teaching system, the availability of remote learning, or cultural differences related to education should also be considered. Therefore, the results of this study cannot be generalized to samples from other countries. Finally, as the SAVE-9 scale was originally developed for COVID-19 frontline healthcare workers, there are some issues with applying this scale to schoolteachers. However, the SAVE-9 scale focuses on individual anxiety or stress regarding a viral epidemic rather than on clinical practice. Therefore, it can be applied to a schoolteacher that cares for students.

In conclusion, like the SAVE-9 scale for healthcare workers, the teachers' version of the SAVE-9 scale is a reliable and valid rating scale for assessing the anxiety and work-related stress of schoolteachers. During the current pandemic, we hope that the SAVE-9 scale will be used to measure the work-related stress and anxiety response of schoolteachers who teach and care for students. We also hope that this scale will assist in the development of support systems that will decrease their psychological distress.

## Data Availability Statement

The raw data supporting the conclusions of this article will be made available by the authors, without undue reservation.

## Ethics Statement

The study protocol was approved and the requirement for written informed consent for participation was waived by the Institutional Review Board of Asan Medical Center (2021-0098). Written informed consent for participation was not required for this study in accordance with the national legislation and the institutional requirements.

## Author Contributions

SC and SS developed the study concept. Testing and data collection were performed by JL, SL, and GJ. Data analysis and interpretation were performed by SC, SS, GJ, and SL. SY and SC drafted the paper. All authors contributed to the study design, provided critical revisions, and approved the final version of the paper for submission.

## Conflict of Interest

The authors declare that the research was conducted in the absence of any commercial or financial relationships that could be construed as a potential conflict of interest.

## Publisher's Note

All claims expressed in this article are solely those of the authors and do not necessarily represent those of their affiliated organizations, or those of the publisher, the editors and the reviewers. Any product that may be evaluated in this article, or claim that may be made by its manufacturer, is not guaranteed or endorsed by the publisher.

## Footnotes

^1^Available online at: https://ourworldindata.org/covid-vaccinations accessed on 2021, July, 12th.

^2^Available online at: https://covid19.who.int/ accessed on 2021, July, 12th.

^3^https://en.unesco.org/covid19/educationresponse accessed on 2021, July, 12th.

^4^http://www.embrain.com.

^5^http://www.save-viralepidemic.net.
